# Recent Advances in Non-Contact Food Decontamination Technologies for Removing Mycotoxins and Fungal Contaminants

**DOI:** 10.3390/foods13142244

**Published:** 2024-07-16

**Authors:** Yan Wang, Aiyun Zhou, Bei Yu, Xiulan Sun

**Affiliations:** 1 College of Food Science and Technology, Zhejiang University of Technology, Hangzhou 310014, China; ay19980305@163.com (A.Z.);; 2 State Key Laboratory of Food Science and Technology, Collaborative Innovation Center of Food Safety and Quality Control, School of Food Science and Technology, Jiangnan University, Wuxi 214122, China

**Keywords:** non-contact food processing technology, fungi, mycotoxin, decontamination

## Abstract

Agricultural food commodities are highly susceptible to contamination by fungi and mycotoxins, which cause great economic losses and threaten public health. New technologies such as gamma ray irradiation, ultraviolet radiation, electron beam irradiation, microwave irradiation, pulsed light, pulsed electric fields, plasma, ozone, etc. can solve the problem of fungal and mycotoxin contamination which cannot be effectively solved by traditional food processing methods. This paper summarizes recent advancements in emerging food decontamination technologies used to control various fungi and their associated toxin contamination in food. It discusses the problems and challenges faced by the various methods currently used to control mycotoxins, looks forward to the new trends in the development of mycotoxin degradation methods in the future food industry, and proposes new research directions.

## 1. Introduction

Mycotoxins are toxic secondary metabolites produced by filamentous fungi under appropriate conditions of temperature and humidity, of which 300–400 molecular species have been identified [1,2]. Mycotoxins are found in many types of food, particularly in grain crops, fruits, vegetables, and milk. The major mycotoxins are aflatoxins (AFs), ochratoxin (OTA), zearalenone (ZEN), deoxynivalenol (DON), T-2 toxin (T-2), and fumonisins (FUMs) [1]. Mycotoxins contaminate foods and enter the food chain, and may pose teratogenic, carcinogenic, neurotoxic, immunotoxic, and genotoxic hazards [2]. Mycotoxins are highly chemically stable, and once present in foods, they are difficult to remove, endangering both human and animal health [3]. Even if the daily intake is very low, cumulative intake of mycotoxins poses a serious threat to the human body. In addition to public health problems, mycotoxins cause significant economic losses to food producers and processors, especially for agricultural products. According to the Food and Agriculture Organization of the United Nations (FAO), more than 25% of the world’s cereals are contaminated with mycotoxins to varying degrees, and the annual losses caused by mycotoxin contamination are as high as billions of dollars, a problem that is particularly serious worldwide [4].

**Figure 1:**
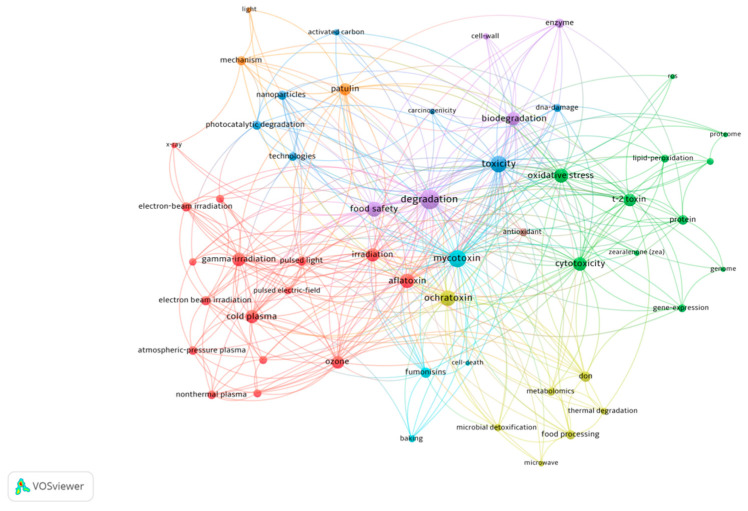
Map of co-occurrence network of keywords by bibliometric analysis. The papers were obtained by searching terms “degradation of mycotoxins” in Web of Science (https://www.webofscience.com/wos/alldb/basic-search, accessed on 5 October 2023).

Based on papers published in the Web of Science core collection from January 2019 to December 2023, on the subject of “degradation of mycotoxins”, a literature-metric analysis of relevant studies was carried out, which identified the main trends in research and potential applications in different research areas (Figure 1). Recently developed processes for degrading mycotoxins mainly involve non-contact food processing or biodegradation. Non-contact food processing technologies, including light irradiation (ultraviolet irradiation, pulsed light), ionization irradiation (gamma rays, electron beams), pulsed electric fields, microwave, plasma, ozone, and some emerging technologies (photocatalytic degradation and nanoparticles), can solve the problem of fungal and mycotoxin contamination which cannot be effectively solved by traditional food processing methods. The main biological process for degrading fungal toxins is microbial or enzymatic degradation. However, these methods have significant limitations, such as difficulty isolating degrading bacteria, low resistance, unstable enzyme activity, and high cost. The bibliometric analysis revealed research trends on the composition and content of mycotoxins in foods and approaches to degrade these mycotoxins. The co-occurrence network map of keywords (Figure 1) indicates that irradiation, plasma, and ozone are the main foci of recent research on processing technology. In particular, gamma ray irradiation, ultraviolet radiation, electron beam irradiation, microwave irradiation, pulsed light, pulsed electric fields, plasma, and ozone are several of the principal non-contact processing methods for the removal of fungi and mycotoxins from food.

**Figure 2:**
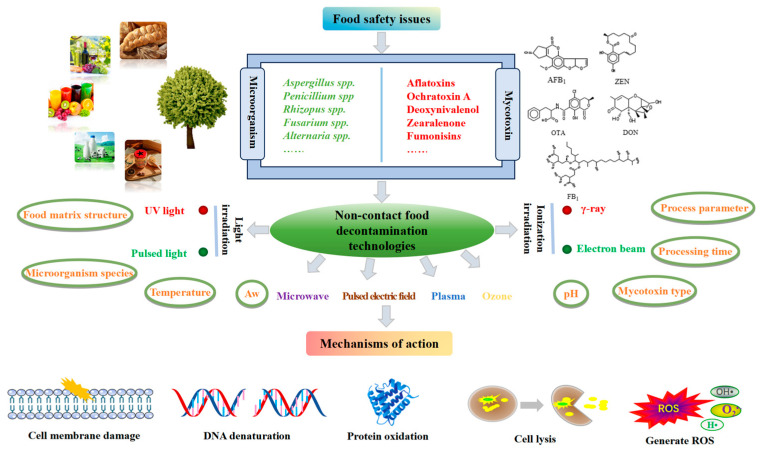
Factors influencing, and mechanisms of action, of non-contact food decontamination methods for removing fungi and mycotoxins.

In recent years, through the continuous exploration of scientific researchers, some non-contact food decontamination technologies have been found to have significant effects, providing a new effective strategy for food decontamination (Figure 2). The effectiveness of food decontamination technology depends on various factors, including the process parameters, time, temperature, pH, water activity, food matrix structure, micro-organism species, and mycotoxin type. There may also be interactions between these factors that influence the effectiveness of degradation methods. The action mechanisms of the non-contact fungal and mycotoxin degradation methods involve the disruption of cell membranes, DNA damage, protein oxidation, cell lysis, or the generation of reactive oxygen species (ROS).

The current status of research on mycotoxin contamination in grains and cereals, nuts and seeds, fruits and vegetables, and milk over the last five years are presented in this review. The main purpose of this review is to introduce several non-contact food decontamination technologies for removing fungi and mycotoxins and summarizes their principles, applications, and factors affecting the decontamination effect.

## 2. Mycotoxins in Food Commodities

### 2.1. Current Status of Research on Mycotoxin Contamination of Grains and Cereals

The quality and safety of grains and cereals have always been a major research focus because they affect the basic food supply and social stability of countries [3]. Fungi and mycotoxins have a major influence on the quality and safety of grains and cereals and contaminate crops both directly and indirectly. The main mycotoxins found in grains and cereals are aflatoxins, deoxynivalenol, zearalenone, ochratoxins, fumonisins, and T-2 toxin [5,6]. Reported levels of mycotoxin contamination in various grains and cereals are summarized in Table 1; these reports show that mycotoxin contamination of grains and cereals is a global problem.

### 2.2. Current Status of Research on Mycotoxin Contamination of Nut and Seed Products

Nuts and seeds are nutritious and unique-tasting food. They are rich in protein, dietary fiber, and healthy fats, which help reduce the risk of many diseases [21,22]. China has a rich variety of nut and seed foods, mainly walnuts, peanuts, almonds, pistachios, and cashews. Nut and seed foods are particularly susceptible to contamination by fungi and mycotoxins due to their production and storage conditions [23]. In nut and seed foods, there are two major categories of common mycotoxins: aflatoxins and ochratoxins (Table 2).

### 2.3. Current Status of Research on Mycotoxin Contamination of Fruits, Vegetables, and Their Processed Products

Fruits and vegetables, as well as their processed products, have a high moisture content, so fungi can easily cause rot and deteriorate during transportation, storage, processing, and marketing [33]. The loss rate of fruits and vegetables during postharvest transportation and storage can range from 35% to 55%, with mycotoxin contamination being the leading cause of this loss [34]. There are five common mycotoxins in fruits and vegetables, namely patulin (PAT), aflatoxins, ochratoxins, alternaria toxins, and trichothecenes [35]. Alternaria toxins include alternariol (AOH), alternariol monomethyl ether (AME), tenuazonic acid (TeA), tentoxin (TENT), etc. Reported levels of mycotoxin contamination of fruits, vegetables, and their processed products are summarized in Table 3. A high occurrence of mycotoxins in different juices and wines has been observed. OTA and PAT were detected the most in apple juice, cava, and cider, while OTA, AOH, and PAT were frequently observed in wine [20].

### 2.4. Current Status of Research on Mycotoxin Contamination in Milk

Milk contains high-quality protein, which is easily digested and absorbed, making it a dietary staple in many parts of the world [40]; milk safety is therefore very important. AFB_1_ is absorbed by cows primarily through the ingestion of contaminated feed and metabolized, then AFM_1_ (Aflatoxin M_1_) is secreted into their milk [41]. Aflatoxin M_1_ is the most common mycotoxin in milk; the reported levels of AFM_1_ contamination of milk are summarized in Table 4. AFM_1_ has been linked to carcinogenesis, cytotoxicity, teratogenicity, and genotoxicity [42]. Infants and the elderly are most susceptible to AFM_1_ toxicity, so they must be protected from consuming contaminated milk.

## 3. Non-Contact Food Decontamination Technology for Removing Mycotoxins

Non-contact food decontamination technology means that there is no direct contact between food and processing equipment or tools during processing. This technology typically uses various forms of energy to process food without the need for physical contact. It is commonly used for food decontamination, sterilization, drying, and other treatments. For example, gamma radiation, ultraviolet radiation, pulsed light, microwave irradiation, plasma, ultrasound, pulsed electric fields, and ozone are non-contact food processing technologies. The difference between non-contact food processing technology and non-thermal processing technology is that the former emphasizes the absence of direct contact between food and processing equipment during processing, while the latter emphasizes that traditional heat treatment is not used. The two are often used in combination to achieve more efficient and environmentally friendly food decontamination effects. Non-contact food decontamination technology can effectively kill or inhibit fungi and degrade their toxins in food, thereby extending the shelf life of food while maintaining the nutrition and flavor of the food. Table 5 shows the latest research results on the removal effect of mycotoxins in food by these technologies.

### 3.1. Gamma Ray Irradiation

#### 3.1.1. Principle

Gamma rays (γ-rays) are a form of very short-wave (<0.01 nm) electromagnetic radiation, emitted by the decay of radioactive isotopes, and are highly penetrating and can degrade DNA, RNA, protein, and other organic molecules. Studies have confirmed that gamma ray irradiation has a significant effect on *Aspergillus conidia* germination and hyphal growth. High radiation doses can cause colonies to die after germination and can also cause mycelium to stop completely in the middle of growth, causing reproductive death [82]. This damage is fatal to micro-organisms but has minimal effects on non-living foods [83]. The hydrogen radical (H^•^), superoxide radical (O_2_^•−^) ,and hydroxyl radical (OH^•^) produced by gamma ray irradiation from reactions with water molecules are highly reactive substances, which can rapidly react chemically with mycotoxins, thereby mediating toxin degradation [84]. For example, up to 97% of OTA in an aqueous solution was degraded by treatment with gamma rays at 8.6 kGy intensity [85], which was attributed to a free radical reaction at the 8, 9 double bond of the furan ring [86].

#### 3.1.2. Applications of Gamma Ray Irradiation

Gamma ray irradiation has the advantages of good sterilization, no residue, no pollution, and so on. Gamma irradiation efficiency is affected by the food matrix, fungal strain, radiation dose, food moisture content, and other factors. Treatment of AFB_1_ in hazelnuts for 10 min with 10 kGy intensity gamma ray irradiation treatment reduced the AFB_1_ content by 47.0% [49]. Treatment of milk samples with 0.39 mGy per day reduced AFM_1_ levels after 4 and 8 days by 51.5% and 99.0%, respectively [50]. A gamma ray irradiation treatment of 3 kGy is sufficient to remove 90% of the natural fungus load in sorghum. Increasing the gamma dose significantly reduced the initial mycotoxin level, and the breakdown rates of AFB1 and OTA in naturally contaminated sorghum reached 59% and 32%, respectively, at a radiation dose of 10 kGy [51]. OTA-containing wheat flour with various moisture contents was treated with γ-ray irradiation; degradation efficiency increased with increasing moisture content and radiation dose, achieving the optimal 24.0% reduction at 30.5 kGy and 32.0% moisture [85]. Similar treatment of grape juice and wine reduced the OTA content by 11.0–23.0%. Degradation of AFB_1_ in peanuts increased from 20.0–43.0% when the γ-ray dose was increased from 5 to 9 kGy [52]. Aflatoxin degradation and the total sugar content in peanuts were greater after irradiation at 10 kGy than at 25 kGy, indicating that mycotoxin degradation does not necessarily increase with increased irradiation intensity [87]. The color, hardness, oil content, peroxide value, and malondialdehyde content of the peanuts did not differ significantly between treatments. Research has evaluated the cytotoxicity of irradiated samples containing ZEA in human hepatocellular carcinoma (HepG2) cells and found that gamma ray irradiation can effectively reduce ZEA, and the cytotoxicity and ZEA estrogenicity was also increasingly reduced with increasing radiation doses [88].

#### 3.1.3. Problems and Challenges

There is a lot of contradicting research on the efficiency of gamma ray irradiation in removing mycotoxins from food. Some studies have found that using gamma ray irradiation can considerably reduce mycotoxins in food and, in many cases, totally eliminate them. However, there are still many studies that show that gamma ray irradiation has limited effectiveness and can even adversely affect food quality. Gamma ray irradiation treatment of mycotoxins in black pepper revealed that ochratoxins and aflatoxins were not totally eliminated, even at a level of 60 kGy [89]. When gamma ray irradiation is used to treat green tea, an appropriate dose can induce the production of new volatile substances, but an excessively high dose will result in an unpleasant taste [90]. Gamma ray irradiation can change the crystal structure of starch, improve its gelatinization and rheological properties, and slow down its digestion rate. However, excessive doses of irradiation have a direct impact on the edible quality of rice, which is not well tolerated by consumers [91]. A high dose of gamma ray irradiation may reduce food quality and produce an irradiation odor [92], while a low amount may fail to achieve the desired decontamination effect. Therefore, strict control of the radiation dose and a quality management system are essential. In addition, gamma rays have strong radioactivity and pose certain risks. During the food irradiation process, some radioactive substances may be produced [93].

### 3.2. Ultraviolet Irradiation

#### 3.2.1. Principle

Ultraviolet light is a form of electromagnetic radiation with a wavelength range of 100–400 nm [94], which is divided into three types, namely long-wave (UV-A; 315–400 nm; the least harmful ultraviolet type), medium-wave (UetV-B; 28–315 nm), and short-wave (UV-C; 200–280 nm) [58]. UV-C can penetrate the cell membrane of micro-organisms, resulting in crosslinking between adjacent thymine and cytosine bases in DNA strands, thereby inhibiting DNA replication and transcription, impairing cell function, and inducing apoptotic cell death [55]. Some scientists have discovered that ultraviolet irradiation can cause apoptosis in fungal cells, alter mitochondrial membrane potential, accumulate intracellular reactive oxygen species, promote lipid peroxidation, and damage cell membranes [95]. Ultraviolet light can also degrade mycotoxins, since most mycotoxins absorb UV light and undergo photocatalytic degradation reactions [96]. Mao et al. used UPLC-TQEF-MS/MS technology to analyze the degradation products of AFB_1_ in peanut oil after ultraviolet irradiation and explored the possible degradation pathways of AFB_1_ under ultraviolet irradiation. Tests on human embryonic liver cell activity showed that the degradation products after ultraviolet irradiation significantly reduced cell toxicity compared to the initial AFB_1_, likely due to the destruction of toxicological sites, and the terminal furan 8,9-double bond of aflatoxin B_1_ was broken [97]. UV-C is the most widely used for removing microbial and mycotoxin contamination from foods [98].

#### 3.2.2. Applications of Ultraviolet Irradiation

UV irradiation is widely used on various food commodities as an effective method of removing fungi and mycotoxins. The effect of UV irradiation on mycotoxins varies depending on the food matrix involved. Treatment of peanut oil with UV-A (365 nm) light for 30 min reduced the content of AFB_1_ by up to 96% [97]; AFB_1_, OTA, and FB_2_ in wheat flour were completely degraded after 15 min of UV-C (254 nm) irradiation [53]. Treatment of corn and peanut samples with UV-C irradiation reduced the AFB_1_ content by 17–43% in maize and 14–51% in peanuts after 10 days of irradiation at 8.37 J·cm^−2^ [54]. During storage, the alternaria toxins content of artificially contaminated tomato samples increased, reaching a peak at 11 days, but the accumulation of mycotoxins was inhibited by 2.5 J·cm^−2^ UV-C irradiation, reducing the AOH, AME, and TeA contents by 44.5, 37.1, and 34.5%, respectively. In addition, the low-dose UV-C irradiation increased the content of some phenolic acids in the tomatoes [55]. UV-A light (0.836 J·cm^−2^) treatment of whole milk reduced the content of AFB_1_ by 78.2%. UV-A light (0.857 J·cm^−2^) treatment of whole milk reduced the content of AFM_1_ by 65.7% [56].

Generally, mycotoxin degradation increases with increasing UV intensity and irradiation time. For instance, the content of AFB_1_ in peanut oil decreased as the irradiation time with UV-C (254 nm) increased; AFB_1_ degradation exceeded 95% after irradiation for 120 s, with no change in the acid, or peroxide values of the oil [57]. Milk samples treated with UV-C (254 nm) for 5, 10, 15, or 20 min showed the highest degradation of AFM_1_, over 50%, after 20 min, regardless of the initial AFM_1_ content [58]. The survival of AFM_1_-treated HepG2 cells increased from 70.42% to 98.44% when the AFM_1_ treatment solution was irradiated with UV-A at 1.2 J·cm^−2^, with no residual aflatoxin toxicity after the UV-A treatment [99]. Nicolau-Lapena et al. reported that in apple juice, patulin degradation exceeded 98% at a UV intensity of 4506 J·cm^−2^. The degradation products were identified by HPLC-MS, and three possible degradation pathways were proposed. Furthermore, the degradation products were found to be less toxic than patulin [59].

The antimicrobial effect of UV rays depends on the micro-organism species and the toxins they produce. For example, UV-C treatment of fungal-infested roasted coffee beans for 2 h decreased *Aspergillus flavus* counts by 2.16 log and *Aspergillus parasiticus* counts by 1.03 log [100]. The color and moisture content of the coffee beans remained unchanged after UV-C irradiation, but there were changes in pH and the acidity.

#### 3.2.3. Problems and Challenges

Ultraviolet radiation can effectively kill micro-organisms on food, thereby extending the shelf life of food. However, ultraviolet radiation can also have some negative effects on the food itself. Firstly, long-term exposure to ultraviolet light can cause food to produce an off-flavor, affecting the taste of the food [101]. Secondly, ultraviolet light can also destroy the nutrients and natural pigments in food, causing it to lose its original color and flavor [102]. In addition, ultraviolet light can also cause lipid peroxidation in food, promoting the production of carcinogens and posing a potential threat to human health [103]. Zhai et al. observed that UVC-LED treatment caused significant changes in the color properties and browning index of orange juice [104]. Research has confirmed that the reason for the change in the browning index of UV-C-treated carrot juice samples may be attributed to the oxidation reaction of certain phytochemicals (polyphenols, carotenoids, etc.) present in the carrot juice during the UV-C application process [105]. Some limitations of ultraviolet irradiation applications, such as short sterilization depth and uneven sterilization, may increase cell survival rates. In order to overcome the adaptive responses of micro-organisms, attempts should be made to synergize ultraviolet radiation with food additives, combine ultraviolet radiation with biological control, etc., as preventive and control methods to enhance the sterilization effect of ultraviolet irradiation for future research and application of ultraviolet radiation technology in food sterilization.

### 3.3. Electron Beam Irradiation

#### 3.3.1. Principle

**Figure 3:**
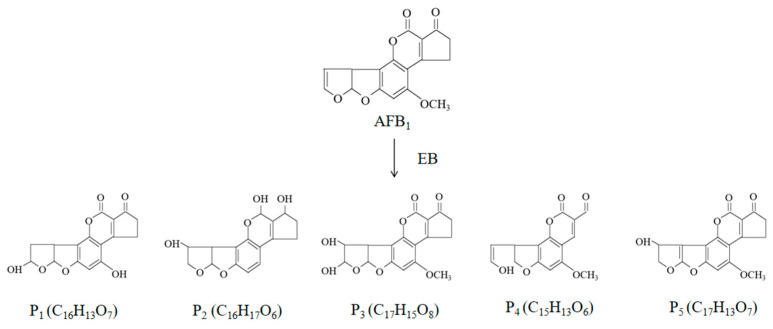
Structural formula of degradation products of AFB_1_ in aqueous media [109]. EB: Electron beam.

Electron beams are produced by an electron accelerator with a specific energy and intensity. Electron accelerators are categorized as high energy (5–10 MeV), medium energy (0.3–5.0 MeV), and low energy (100–300 KeV) [106]. Their mechanism of action is comparable to that of gamma rays, degrading DNA and other macromolecules in living cells and degrading mycotoxins by directly generating highly active radicals, such as hydrogen and hydroxyl radicals, by a homolytic splitting of water molecules [107]. Aflatoxin degradation by electron beam irradiation is much more effective in the presence of water [108]. Electron beam irradiation treatment decomposes aflatoxin B_1_ in an aqueous solution to produce five degradation products (Figure 3), four of which lacked the terminal furan 8,9-double bond of aflatoxin B_1_ [109]. This double bond is essential for the carcinogenicity and toxicity of aflatoxins and mediates interactions with DNA and proteins. The electron beam irradiation degradation products of aflatoxin B_1_ lost this double bond because of changes to the furofuran ring, lactone ring, cyclopentenone ring, or the methoxyl group [110].

#### 3.3.2. Applications of Electron Beam Irradiation

Electron beam irradiation is widely used in food processing because of its convenience, economy, and safety. Electron beam irradiation in the range of 1–10 kGy was used to degrade zearalenone and vomitoxin; as the irradiation intensity increased, toxin degradation increased [111]. Degradation of 1 μg·mL^−1^ zearalenone and vomitoxin at 10 kGy intensity was by 76.04 and 89.31%, respectively. Corn contaminated with ZEN and OTA was treated with 50 kGy electron beam irradiation; degradation of ZEN and OTA were 71.1 and 67.9%, respectively [112]. ZEN and OTA-contaminated corn causes liver lesions in mice that eat the corn, but electron beam irradiation of the corn effectively reduced mycotoxin hepatotoxicity [113]. After electron beam irradiation treatment, the content of OTA in red pepper varied from 118.1 to 156.7 μg·kg^−1^ and decreased with the increase in irradiation dose; at a dose of 30 kGy, the OTA content was reduced by 25% [60]. The efficiency of AFB_1_ degradation in peanut meal by electron beam irradiation was primarily related to the initial AFB_1_ concentration and the moisture content [114]. Degradation of 5 μg·mL^−1^ AFB_1_ in peanut meal with a moisture content of 21.47% was faster than that of 1, or 0.5 μg·mL^−1^ AFB_1_, with moisture contents of 14.32% and 8.74%, respectively. The cytotoxicity of the degradation products of AFB_1_ in peanut meal was much less than the original AFB_1_. Corn syrup containing aflatoxin and fumonisin was treated with electron beam irradiation (20 kGy); the aflatoxin content decreased by 0.3 log (ng·g^−1^) on average, but the fumonisin content did not change significantly [61]. Electron beam irradiation (16 kGy) of ZEN and OTA in methanol and acetonitrile reduced the ZEN content by 92.76 and 72.29%, respectively, and that of OTA by 84.16 and 91.56%, respectively [115].

#### 3.3.3. Problems and Challenges

Although electron beam irradiation can effectively inhibit micro-organisms, the electron penetration rate is low, and the sterilization effect is affected by factors such as the type of food and packaging. In addition, high doses of electron beam irradiation may have adverse effects on food quality, and different types of food have varying degrees of tolerance to electron beam irradiation [116]. Electron beam irradiation can also affect the flavor of food. Some researchers believe that meat treated with electron beam irradiation can produce an odor that consumers dislike. The production of this off-odor may be due to the cleavage or cross-linking of sulfur-containing proteins in the meat after electron beam irradiation, resulting in the production of compounds with bad odors, such as methanethiol and hydrogen sulfide, which could be due to the oxidation and decomposition of lipids by electron beam irradiation, which produces some off-flavor compounds [117]. Research by Arshad et al. indicates that electron beam irradiation has a detrimental effect on the rate of lipid oxidation in meat [118]. Currently, electron beam irradiation technology has been widely used in food sterilization, preservation, and shelf life extension. In the future, the parameters of electron beam irradiation treatment should be continuously optimized to significantly extend the shelf life of food while better maintaining product quality.

### 3.4. Microwave Irradiation

#### 3.4.1. Principle

Microwaves are a form of electromagnetic radiation with a wavelength range of 1 mm–1 m and a frequency range of 0.3–300 GHz [119]. Microwave irradiation treatment has the potential to eliminate fungal and mycotoxin contamination and is an emerging food processing method [120]. Microwave irradiation treatment kills micro-organisms in food through thermal (heating) and non-thermal effects; non-thermal effects are chemical or biochemical changes that do not occur at the same temperature as other heating methods. There appears to be a synergistic interaction between the two effects, i.e., the higher the temperature, the stronger the synergy [121]. However, the existence of the non-thermal effect is controversial. The thermal effect of microwave irradiation treatment of food appears to be dominant [122]; the mechanism of microwave heating involves interactions of polar molecules and ions with the microwave alternating electric field, which generates heat [120]. Microwave heating can degrade proteins, enzymes, and nucleic acids, change the calcium ion permeability of the microbial membrane, and increase water loss from microbial cells, which can inhibit growth or induce cell death [123]. Mycotoxins degradation works on the idea of using microwave energy to fast-rotate molecules and create a significant bombardment effect, lowering the strength of chemical bonds and breaking some chemical bonds to achieve the goal of degradation. Zhang et al. discovered that the difuran ring double bond group in the AFB_1_ structure vanished following microwave irradiation treatment through chemical structural analysis [124].

#### 3.4.2. Applications of Microwave Irradiation

Microwave irradiation technology is now widely used, not only for food sterilization but also for various other food processing applications. The degradation of AFB_1_ in peanuts was 62, 67, and 59% when microwaved at 600 W (3 min), 480 W (5 min), and 360 W (6 min), respectively [52]. *Aspergillus parasiticus* and *Aspergillus flavus* spores microwaved at 50 °C for 5 min decreased in number by 1.16 and 1.45 log, respectively [125]. Similarly, microwave irradiation treatment for 20 s reduced the spore counts of *A. parasiticus* on brown rice and barley by 1.20 and 1.00 log, respectively, and those of *A. flavus* by 1.06 and 1.05 log, respectively [5]. After 15 s of microwave irradiation treatment, *Fusarium spp.* and *Microdochium nivale* in wheat decreased by 72% and 77%, respectively [126]. The DNA levels of both fungi in wheat were unchanged after microwave irradiation treatment, implying that cell death was caused by the microwave thermal effect rather than DNA damage. Degradation of mycotoxins depends on microwave heat treatment time; AFB_1_ and OTA in maize flour were reduced by 50.58% and 46.97%, respectively, after heating for 10 min [62]. The content of aflatoxin B_1_, B_2_, G_1_, G_2_, and OTA in pistachio nuts decreased by 34.6%, 23.3%, 29.3%, 36.6%, and 34.2%, respectively, after 10 min of microwave irradiation treatment [63]. Microwave irradiation reduces the fungal cell counts in treated samples, and the reduction is related to the irradiation dose. For example, 2400 W irradiation failed to reduce fungal cell counts in almond samples, but 3000 and 4000 W irradiation significantly reduced fungal cell counts; the cell count reduction was maintained after six months of storage [127].

#### 3.4.3. Problems and Challenges

Microwave irradiation treatment has advantages such as high efficiency and strong controllability, and it is widely used in food. However, issues such as poor uniformity of temperature distribution and unstable product quality in microwave heating have limited the application of microwave irradiation technology in industrialization. The combined effect of different electric field distributions in the microwave cavity and differences in material heat transfer capabilities results in cold and hot zone distributions in microwave heating [128]. The “cold spots” lead to incomplete inactivation of micro-organisms in food, posing a risk of potentially pathogenic micro-organism revival; the “hot spots” cause food color degradation, reducing product quality [129]. At the same time, research has found that pigment degradation, protein denaturation, enzymatic browning, and non-enzymatic browning are the main reasons for the color change in food caused by microwave irradiation treatment [130]. The distribution of the electric field and the unevenness of microwave irradiation treatment can be mitigated by changing the position of the sample being treated and optimizing microwave process parameters.

### 3.5. Pulsed Light Irradiation

#### 3.5.1. Principle

Pulsed light sterilization treats the food surface with an intense pulse of broad-spectrum white light (wavelength 200–1100 nm, including ultraviolet, visible and infrared) [131]. Pulsed light is produced by a high-power xenon lamp with a high-voltage DC power supply and a cooling system [132]; the pulse has a high intensity over a very short time (tens to hundreds of microseconds). Pulsed light is an effective way to inactivate micro-organisms and degrade mycotoxins on food surfaces. It involves photothermal photochemical, and photophysical effects [131]. The visible and near-infrared components of the light pulse heat the food surface, damaging the cell structure of micro-organisms and causing cell death, whereas the ultraviolet component damages DNA by forming pyrimidine dimers, resulting in mutations, inhibition of DNA replication, and cell death [133]. Transcriptomic analysis of pulsed light inhibition of *Aspergillus carbonarius* growth detected negative effects on DNA replication, glucose metabolism, cell integrity, and secondary metabolism [134]. Pulsed light can also degrade mycotoxins; for example, AFB_1_ degradation appears to involve cleavage of the terminal furan double bond and opening of the lactone ring [135].

#### 3.5.2. Applications of Pulsed Light Irradiation

Pulsed light irradiation is widely used in the food industry because of its high efficiency, low energy consumption, and safety. *A. niger* and *A. flavus* counts on barley grains decreased by 1.2 log and 1.7 log, respectively, when treated with pulsed light for 15 s at 18.0 J·cm^−2^ [136]. The capacity of *Botrytis cinerea* forming colonies on agar medium was inhibited by pulsed light, and flow cytometry revealed that it damaged the membrane. It manifests as the cell wall detaching from the plasma membrane, the cytoplasm collapsing and vacuolizing, the cell wall and plasma membrane rupturing, the loss of a considerable amount of cytoplasm, and even organelle rupture [137]. The surface temperature of peeled peanuts reached 178 °C after pulsed light irradiation treatment, reducing the AFs (AFB_1_ and AFB_2_) content by up to 91% [135]; the AFs content of unpeeled peanuts was reduced by 82%, by the same treatment, indicating that the peanut skin reduced the effectiveness of pulsed light. In addition, the higher the moisture content of the peanuts, the greater the degradation of AFs. The reduction in AFs content after 5 min was greater at 16% moisture than at 10% and 4%, with differences of ~31% and 70%, respectively. Wang et al. investigated the degradation of AFs in rice bran by pulsed light irradiation; the contents of AFB_1_ and AFB_2_ decreased by 90.3% and 86.7%, respectively, after 15 s of pulsed light irradiation [138]. The DON content of ungerminated barley decreased by 30.9% after 180 light pulses; the presence of the barley husk, which blocks the effect of pulsed light irradiation , may explain the relatively slow degradation of DON [64]. Degradation of OTA in grape juice by pulsed light irradiation was most effective, reaching 95.18% at a pulse intensity of 39 J·cm^−2^ [65]; there was no significant difference in pH, soluble solids, total organic acids, or color between treated and untreated grape juice. Fungal community analysis of Chinese bayberries treated with pulsed light indicated that the treatment effectively reduced the relative abundance of pathogenic fungi [139]. Patulin degradation in apple juice increased with the pulsed light dose; patulin degradation was 74% at 24 J·cm^−2^ [66]. Different initial patulin concentrations in apple juice had no significant effect on patulin degradation by pulsed light irradiation at 40.50 J·cm^−2^, achieving 96.27% degradation at 0.1 μg·mL^−1^ and 95.75% at 0.5 μg·mL^−1^ [131]. Degradation of aflatoxins decreased as the distance between the sample and the xenon lamp increased [68]. Degradation of aflatoxins in apple juice increased with the number of pulses, with 20 μg·mL^−1^ AFB_1_, AFB_2_, AFG_1_, and AFG_2_ in apple juice reduced by 71.96%, 73.32%, 54.04%, and 69.58%, respectively, after 40 pulses.

#### 3.5.3. Problems and Challenges

Although pulsed light irradiation has significant advantages in food decontamination, its practical application is influenced by various factors, such as the type of food, initial bacterial load, pulse flux, etc. There are still certain limitations and negative effects when pulsed light irradiation is used in food sterilization. Pulsed light irradiation is a surface sterilization technology that has good sterilization effects on solid surfaces and transparent liquids. Due to the presence of phenomena such as light shielding, reflection, refraction, and scattering, the sterilization effect of pulsed intense light on dark-colored liquids or uneven surfaces is relatively poor. After treatment with a higher dose or longer duration of pulsed light, adverse effects on food quality may occur [140]. After treatment with pulsed light irradiation at 12.81 J·cm^−2^, the pork developed a pungent odor, and it also affected the oxidation of proteins and lipids in the meat [141]. In 1996, the FDA stipulated that the pulse flux applied to food should not exceed 12 J·cm^−2^. In fact, the pulsed flux used in most studies has already exceeded this standard, and there is still no clear regulation on the range of flux on the food surface. At present, more comprehensive research and application cases are still needed to provide strong technical support for the application of pulsed strong light technology in the food industry.

### 3.6. Pulsed Electric Field

#### 3.6.1. Principle

Pulsed electric field (PEF) is a new type of food processing technology that uses a high-voltage, short-term pulsed electric field to treat food, which can kill micro-organisms, degrade mycotoxins, improve food quality, and extend food shelf life [4,142]. The working principle of the pulsed electric field for killing micro-organisms is to use high-voltage pulses generated by a pulse power supply to apply an instantaneous, strong electric field to microbial cells, disrupting the structure and function of the cell membrane, leading to cell death or inactivation [143]. The action mechanism of PEF for degrading mycotoxins mainly includes two aspects: one is the direct effect that the high voltage and short electrical pulses generated by the electric field on mycotoxin molecules, resulting in their chemical bond breaking or redox reaction; the other is the indirect effect of the electric field on the cell or matrix where the mycotoxins are located, which changes its physical properties or biological activity and promotes mycotoxins degradation [4]. The benefits of a pulsed electric field include the ability to treat food at room temperature or low temperatures, avoiding the loss of nutrition and flavor caused by heat treatment [144].

#### 3.6.2. Applications of Pulsed Electric Field

The antimicrobial effect of PEF is related to factors such as electric field strength, pulse width, microbial species, treatment time, and temperature. Bulut et al. used pulsed electric field (PEF) treatment on sesame seeds to investigate sesame quality parameters; the amount of *Aspergillus parasiticus* dropped by 60% when the PEF energy was 17.28 J [69]. The content of AFB_1_, AFB_2_, AFG_1_, and AFG_2_ dropped by 86.9%, 98.7%, 94.7%, and 92.7%, respectively. The peroxide value of sesame seeds was reduced by 67.4% after PEF treatment, the acid value was lowered by 85.7%, and the color L*, a*, b*, and hue values remained essentially intact. The maximum inactivation efficiency of the number of *Aspergillus parasiticus* on red pepper was 64.37% when the PEF energy was 17.28 J. Moreover, the concentrations of AFB_1_, AFB_2_, AFG_1_, and AFG_2_ in red pepper decreased by 97.75%, 99.58%, 99.88%, and 99.47%, respectively [72]. After PEF treatment, the content of mycotoxins (enniatins (ENS) and beauvericin (BEA)) in juices and smoothies degraded between 43% and 70% [70]. The degradation rates of AFB_1_, AFB_2_, AFG_1_, and AFG_2_ in grape juice were 25%, 72%, 84%, and 24%, respectively, under PEF with 30 kV voltage, 3 kV·cm^−1^ field strength, and 500 kJ·kg^−1^ specific energy [71]. Stranska et al. used two different intensities of pulsed electric fields to treat mycotoxins in malting barley. The content of DON was reduced by 14% and 31%, respectively, and T-2 by 18% and 24% at the lower- and higher-strength PEF, respectively [73].

#### 3.6.3. Problems and Challenges

Compared with foreign countries, most of the research on pulsed electric field technology in China is still in the laboratory research stage or pilot stage, and there is a certain gap from industrialization. Although pulsed electric field technology has great advantages in maintaining the flavor and nutritional components of food with its low thermal effects, it also has certain technical defects. For example, it is difficult to completely kill pathogenic micro-organisms, and high sterilization intensity can easily cause high temperatures; the investment and operation costs may be higher compared to other methods; excessive electric field intensity can reduce the life of high-voltage pulse sources, easily cause electrode corrosion, and cause product pollution, etc. To solve the above problems, in-depth research and technological innovation are needed [145,146].

### 3.7. Cold Plasma

#### 3.7.1. Principle

**Figure 4:**
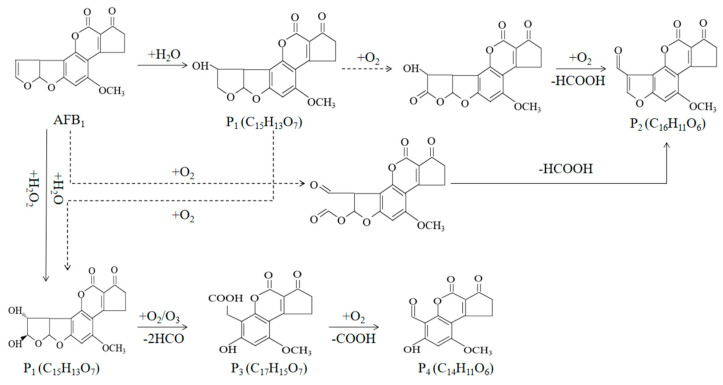
Proposed cold atmospheric pressure plasma mediated degradation pathways of AFB_1_ [152].

Plasma is the fourth form of matter that exists in addition to solid, liquid, and gas. It is mostly made of photons, ions, and free radicals (such as reactive oxygen and nitrogen), and it has distinct physical and chemical properties [147]. The circumstances for producing low-temperature plasma are relatively modest, and it is classified as cold plasma and thermal plasma. Cold plasma is widely acknowledged to be non-thermal and can achieve high inactivation efficiency when applied to food surfaces [148]. Cold plasma can have an effect on the fungal spore cell membrane, and electroporation and etching caused by the active material of the plasma can completely disintegrate the fungal spore membrane [149]. Studies have shown that reactive nitrogen and oxygen species (RONS) generated in cold plasma are effective antimicrobial agents that can degrade a variety of toxic compounds, including mycotoxins [150]. The principle of cold plasma degradation of mycotoxins is mainly to use active species in plasma, such as electrons, ions, free radicals, ultraviolet rays, etc., to react with mycotoxin molecules, destroy their chemical structure, and thereby reduce their toxicity [151]. Hojnik et al. used non-equilibrium plasma to target the degradation of AFB_1_, a method that achieved 100% decontamination in less than 120 s of treatment time [152]. Studies have shown that breaking the vinyl bond between the 8- and 9-positions on the terminal furan ring of AFB_1_ quickly is crucial for inhibiting toxicity. Through ultra-performance liquid chromatography/quadrupole time-of-flight-tandem mass spectrometry (UPLC-qTOF MS) analysis, it was found that the tandem mass spectrometry (MS/MS) cleavage of AFB_1_ consisted of the progressive cleavage of carbonyl groups, which was mainly divided into three steps: (1) double cleavage of carbonyl groups and loss of methyl groups resulted in fragment ions with m/z of 285 and 257; (2) sequential loss of carbonyl groups and methane; (3) carbonyl group recleavage, complementary to the cleavage that happens in the first step (Figure 4). Existing data also demonstrated that high-voltage atmospheric cold plasma (HVACP) could effectively degrade AFM_1_ in milk, and the C8-C9 double bond in the furan ring is removed in the degradation product [153].

Many aspects influence the plasma’s ability to destroy mycotoxins, including plasma type, parameters, reaction time, temperature, operating gas composition, catalyst, and so on [142].

#### 3.7.2. Applications of Cold Plasma

Devi et al. used cold plasma technology to sterilize peanut samples. The results showed that after 12 min of cold plasma treatment under the condition of 60 W power, the AFB_1_ content in peanut samples decreased by more than 95% [149]. The AFB_1_ C8-C9 double bond would be broken during HVACP treatment, resulting in the loss of AFB_1_ toxicity [154]. Ott et al. confirmed that HVACP treatment can inactivate fungal spores and mycotoxins in a short time, with approximately 50% of *Aspergillus flavus* spores inactivated after 1 min of HVACP treatment. After 20 min of HVACP treatment of DON aqueous solution (100 μg·mL^−1^), the degradation rate of DON reached more than 99%, and more than 80% of Caco-2 cell viability was rescued [155]. Shi et al. studied the sterilization effect of cold plasma technology on corn with varied humidity levels. They discovered that after 10 min of cold plasma therapy, the degradation rate of aflatoxins in maize with a relative humidity of 40% could reach 82% [156]. Aflatoxin is degraded more effectively in moderately damp maize treated by cold plasma than in dry samples (5% RH). Hojnik et al. used atmospheric pressure air plasma technology to study the decontamination effect of mycotoxins. After 480 s of treatment, they discovered that the content of AFs, T-2 toxin, FB, and ZEN decreased by 93%, 90%, 93%, and 100%, respectively [157]. Kis et al. found that the degradation efficiency of T-2 and HT-2 in oat flour is dependent on the kind of gases utilized to generate the plasma [74]. The treatment of oat flour with low-pressure dielectric barrier discharge (DBD) plasma created by the ionization of four working gases (nitrogen, oxygen, air, and argon) had a significant effect on the breakdown of T-2 and HT-2 toxins. The breakdown rates of T-2 and HT-2 toxins after 30 min of nitrogen exposure were 43.25% and 38.54%, respectively. Fresh wolfberries are prone to *Alternaria alternata* contamination. After DBD treatment, the maximum reduction of *A. alternata* was 2.26 log CFU/mL, according to the data [158]. At the same time, DBD destroyed the integrity of the *A. alternata* cell membrane while increasing its permeability. ROS generated by DBD accumulate reactive oxygen metabolites in *A. alternata* spores and limit the function of antioxidant enzymes. Guo et al. treated rice grains artificially infected with molds or mycotoxins with cold plasma (CP) [76]. After CP treatment, the microbial activities of *Aspergillus niger*, *Rhizopus oryzae*, and *Fusarium graminearum* were significantly suppressed, and the concentrations of DON and OTA decreased by 61.25% and 55.64%, respectively. Cold atmosphere plasma (CAP) is a potential approach for fungi and mycotoxins control, and the effects of CAP in controlling *Fusarium* spp. contamination on cereals was studied. The findings of the experiments revealed that 3 min CAP achieved effective inactivation (2 to 6-log reduction) of *Fusarium* spore. The various RONS created by CAP in water killed four *Fusarium* strains by damaging cell membranes, accumulating intracellular ROS, and depolarizing mitochondrial membranes [159]. At the same time, CAP efficiently inhibited DON biosynthesis by suppressing acetyl-CoA production, toxisomes formation, and the expression of key trichothecene biosynthetic gene (*TRI*). The degradation rate of DON in aqueous solution after 25 min of treatment reached 98.94% under the condition of double DBD-CP with voltage 100 V and frequency 200 Hz and 61% in wheat after 15 min [75]. Existing data demonstrate that high voltage atmospheric cold plasma (HVACP) effectively degrades aflatoxin M_1_ in milk. The toxicity of the AFM_1_ samples treated with HVACP was reduced as a result of the removal of the C8-C9 double bond in the furan ring in the degradation product [153]. Nguyen et al. found that the removal effect of HVACP on AFM_1_ in skimmed milk varied with the operating gas [77]. When gas containing 65% oxygen was used as the operating gas, the degradation rate of AFM_1_ in milk increased significantly from 38.5% when air was the operating gas to 78.9%. Wielogorska et al. reported that by fitting the first reaction order kinetics and determining the half-lives of each mycotoxins, they discovered that mycotoxins with fat chains decomposed faster [160]. Simultaneously, it was confirmed that more compact aromatic ring structures boost mycotoxin’s structural stability, thereby decreasing the breakdown rate of plasma-induced mycotoxin. They tested the toxicity of the degradation products of aflatoxin B_1_ after low-temperature plasma treatment on HepG2 cells, and no increase in cytotoxicity was observed.

#### 3.7.3. Problems and Challenges

Plasma treatment is an emerging, environmentally friendly sterilization technology. However, in practical applications, plasma sterilization technology still has limitations in terms of process and technology. Plasma treatment is relatively expensive, which increases the cost of production and processing to a certain extent. Plasma treatment has a poor deep sterilization effect on thick food [161]. If improperly handled, it will produce a large amount of active oxygen substances such as ozone, superoxide anions, and nitric oxide, thereby affecting the color and pH of the food, promoting the oxidation of food lipids, and having a negative impact on its flavor [162]. Free radicals generated during plasma treatment facilitate the production of aldehydes, alcohols, ketones, acids, and other small-molecule flavoring compounds from lipid oxidation, which are secondary oxidation products. These can create fishy, metallic, or even foul smells, thereby reducing food quality [163]. After the beef loin is treated with plasma for 10 min, its TBARS value significantly increases, accompanied by the production of a rancid odor, leading to a decrease in the overall sensory evaluation [164]. There are currently just a few investigations on the toxicity of mycotoxin breakdown products after treatment with low-temperature plasma technology. It is impossible to fully prove the safety and reliability of plasma treatment, and additional toxicity studies are required.

### 3.8. Ozone

#### 3.8.1. Principle

The ozone molecule is a colorless, odorless gas made up of three oxygen atoms [165]. Due to the powerful oxidability, ozone may effectively reduce the number of molds in agricultural products during storage and decompose mycotoxins produced by mold, considerably enhancing agricultural product storage time [166]. By progressively oxidizing key biological components, ozone can inactivate micro-organisms through two ways. The first is to promote cell death or disintegration by oxidizing unsaturated lipids in the cell membrane, resulting in the leakage of cell contents and, finally, microbial lysis. The other Is the oxidation of cellular proteins, amino acids of enzymes, and sulfhydryl groups, which results in cell death [167]. As a strong oxidant, ozone can also degrade mycotoxins by disrupting the structure of mycotoxins and altering biological activities [168]. Ozone primarily operates by breaking double bonds in their structure and altering in less dangerous compounds [169]. For example, ozone degrades aflatoxin by attacking the C8-C9 double bond on the furan ring, releasing aldehydes, ketones, acids, and carbon dioxide [170]. Moreover, ozone is unstable and spontaneously converted into oxygen when heated; it does not leave toxic residues on agricultural products, and the FDA has approved it as a safe and effective antibacterial agent in food processing.

#### 3.8.2. Applications of Ozone

At the moment, ozone is primarily used in agricultural products in gaseous and liquid form [171]. Broccoli is sensitive to *Alternaria* infection during cultivation, and the incidence of *Alternaria* in broccoli was reduced from 18% to 2~3% after ozone water treatment. At the same time, ozonated water can improve the quality and nutritional value of broccoli [172]. Torlak tested the potential of gaseous ozone to degrade OTA in sultanas and found that after 120 and 240 min of continuous exposure to gaseous ozone in sultanas, the concentration of OTA in sultanas was reduced by 60.2% and 82.5%, respectively [78]. Simultaneously, gaseous ozone treatment reduced the naturally occurring fungal community on sultanas by almost 2.2 log.

Ozone has been shown to destroy major mycotoxins found in agricultural goods such as AFs, OTA, DON, ZEN, and FB. da Luz et al. discovered that after 5 h of exposing parboiled rice to ozone, the levels of AFB_1_, AFB_2_, AFG_1_, and AFG_2_ in parboiled rice were reduced by 80.9%, 59.2%, 61.8%, and 47.6%, respectively [80]. Among them, the content of AFB_1_ and AFG_1_ showed the largest reduction, which may be due to the ozone attack on the double bond of C8-C9 and the production of many intermediate products, resulting in a greatly reduced toxicity of ozone decomposition products. DON, OTA, and ZEA were decreased by 56.0%, 87.9%, and 75.9%, respectively, after 5 h of ozone exposure. The mechanism of DON breakdown by ozone treatment is that the double bond on C9-C10 is first broken. Then, it reacts with intermediate molecules until it is totally oxidized, forming simple chemicals such as acids, aldehydes, ketones, and so on. The electrophilic addition of double bonds is the primary mechanism by which ZEA and OTA degrade after ozone treatment. Yang et al. found that 50 μg·mL^−1^ ZEN standard solution treated with 2.0 mg·L^−1^ ozone was not detected in the solution after 10 s. They reported that the degradation rate of the OTA standard solution with a concentration of 5 μg·mL^−1^ was 34% after 180 s of ozone treatment with 50 mg·L^−1^ for 180 s [115]. The degradation rate of ZEN and OTA standard operating solutions increases as the duration of ozone treatment increases, and ozone has a greater degradation effect on ZEN. Ribeiro et al. applied ozone at a concentration of 13.5 mg·L^−1^ and a flow rate of 1 L·min^−1^, and after 24 h of ozone treatment, the quantities of FB_1_ and FB_2_ in corn kernels were reduced by 81.2% and 86.2%, respectively [81]. In one study, ozone was used to degrade patulin in water, and the cytotoxicity of ozone degradation of patulin on HepG2 cells was investigated. Patulin was treated with 10.60 mg/L ozone at a flow rate of 90 s per minute, resulting in a 59.94% disintegration rate. Following 90 s of ozone detoxification, the survival percentage of HepG2 cells increased considerably, from 42.31% to 93.96% [173].

The efficiency and conditions of ozone degradation of mycotoxins are affected by many factors, such as ozone concentration, reaction time, temperature, pH value, moisture content, etc. [174]. The ozone concentration of 2.42, 4.38, 8.88, and 13.24 mg·L^−1^ was used to treat Brazil nuts. When the ozone concentration was 2.42 and 4.38 mg·L^−1^, the number of *A*. *flavus* on the nuts decreased by 1.39 and 1.89 log (CFU·g^−1^), respectively, after 240 min of treatment, while when the concentrations of 8.88 and 13.24 mg·L^−1^ were used, the reduction in *A. flavus* was greater than 2.80 log (CFU·g^−1^) after only 60 min of treatment [175]. Santos Alexandre et al. discovered that the initial average concentration of ZEN in whole corn flour was 973 ± 63 g·kg^−1^, which decreased rapidly after 51.5 mg·L^−1^ ozone treatment, and the concentration of ZEN decreased significantly by 37.9% after 5 min of treatment [79]. ZEN degradation reached 62.3% after 60 min of ozonation. Li et al. used ultraviolet ozone to cure intentionally contaminated peanuts, with ozone concentrations of 3, 5, and 7 mg·L^−1^ and irradiation periods of 10, 20, 30, and 60 min, respectively [176]. The degradation rates of AFB_1_, AFB_2_, AFG_1_, and AFG_2_ increased with increasing irradiation time and ozone content. The degradation rates of AFB_1_ and total AF_S_ in peanuts were 79.01% and 67.24%, respectively, after 30 min of 5 mg·L^−1^ ozone and UV irradiation. There was no discernible difference in the amount of polyphenols, acid value, or peroxide in peanuts when compared to the control group. According to the studies mentioned above, using ozone to decontaminate agricultural goods holds a lot of promise, especially when paired with other technologies.

#### 3.8.3. Problems and Challenges

The use of ozone in the food industry is becoming increasingly widespread, as it can control or reduce microbes and extend the shelf life of food. However, ozone also has its disadvantages in practical use. For instance, ozone is unstable and degrades over a short time. Ozone is toxic, and when its concentration is too high, it can harm the human body, so its concentration needs to be controlled during use. At the same time, the cost of ozone production is high, and the yield is low [177].

## 4. Conclusions

Food contamination by fungi and their mycotoxins is a major challenge; food safety is closely related to public health, and fungal contamination of food is a serious threat to both. The non-contact food decontamination methods have the advantages of effectiveness, energy efficiency, and freedom from residues compared with conventional methods. Emerging decontamination methods, such as gamma ray irradiation, ultraviolet irradiation, electron beam irradiation, pulsed light irradiation, microwave irradiation, plasma, pulsed electric field, and ozone, have been extensively evaluated for sterilization of various foods contaminated with fungi and degradation of the mycotoxins the fungi produce. Different decontamination methods are best suited to different types of food. Generally, ultraviolet irradiation and pulsed electric fields are more effective for decontaminating liquid products, whereas pulsed light irradiation, plasma, and ozone are more effective for fruit, vegetables, and their processed products. Gamma- and electron beam-irradiation are more effective against fungi and mycotoxins in grains and cereals. The use of these methods must balance conflicting requirements, i.e., maximizing the killing of fungal cells and spores and the degradation of mycotoxins while minimizing damage to the nutritional and sensory quality of the treated food. In the future, the following issues should be addressed in relation to the degradation of mycotoxins in food: (1) explore the comprehensive treatment technology combining non-contact methods and a variety of novel methods to achieve higher detoxification efficiency and enhance adaptability to different food matrices; (2) while focusing on reducing mycotoxin content in food, it is also necessary to consider food quality and nutritional value; and (3) investigate the mechanism of degradation methods, identify and analyze degraded products, and assess the toxicity of degradation products using cell and animal experiments.

## Figures and Tables

**Table 1:** Contamination of mycotoxins in grains and cereals.

Product	Type of Mycotoxin	Detection Rate (%)	Contamination Value (μg/kg)	Country	Reference
Corn	FB_1_ FB_2_	40.7 37.0	average 1559.00 average 278.00	Spain	[7]
	AFB_1_	100.0	1.69~403.00	Kenya	[8]
	FB_1_ T-2 ZEN	96.7 100.0 23.3	289.00~4243.00 24.60~25.70 20.40~579.00	Algeria	[9]
Wheat	DON ZEN	59.8 18.6	ND~955.00 ND~300.00	Romania	[10]
	T-2 DON ZEN	100.0 90.0 63.3	16.60~47.20 68.30~1363.00 9.60~295.00	Algeria	[9]
Rice	AFB_1_	4.8	ND~6.28	China	[11]
	AFs OTA	56.0 72.0	0.05~21.40 0.03~80.70	Turkey	[12]
	OTA CIT	6.3 13.3	8.00–25.00 49.00–92.00	India	[13]
Barley	DON AFB1 ZEN OTA T-2	44.4 1.4 2.8 8.3 8.3	ND~6880.00 average 26.50 ND~962.00 ND~127.00 ND~56.80	Canada	[14]
	DON	20.0	138.00~973.00	Turkey	[15]
Cereals	AFs	72.0	0.18~25.93	Ghana	[16]
	DON	56.4	average 6.18 maximum 912.29	China	[17]
Sorghum	FUMs	25.0	6.00–16.00	Togo	[18]
	AFs FUMs OTA	100.0 50.0 72.0	average 29.97 average 3269.80 average 37.50	Niger State of Nigeria	[19]
Beer	DON AFB_1_	80.0 60.0	0.87–10.60 8.44–11.82	Spain	[20]

ND: Not detected; CIT, Citrinin.

**Table 2:** Contamination of mycotoxins in nut and seed products.

Product	Type of Mycotoxin	Detection Rate (%)	Contamination Value (μg/kg)	Country	Reference
Peanuts	AFs	76.0	average 37.94	Uganda	[24]
	AFB_1_	92.0~100.0	average 6.37	Tanzania	[25]
	AFB_1_ AFB_2_ AFG_1_ AFG_2_	13.3	1.90~6.50 1.80~2.90 9.60~23.80 5.50~13.20	Morocco	[26]
Coffee beans	AFB_2_	28.3	1.40~87.90	Tunisia	[27]
	AFG_1_	73.6	18.30~145.00		
	AFG_2_	66.0	28.70~218.20		
	OTA	13.2	10.70~122.60		
	ZEA	51.0	25.40~231.70		
	AOH	22.6	109.70~321.00		
	TENT	98.0	11.20~286.00		
	OTA	7.5	0.19~1.12	Cambodia	[28]
Almond	AFB_1_	18.0	1.00~15.50	Italy	[29]
	AFs	22.0	1.20~15.30		
Pistachios	OTA	18.0	>5.00	California	[30]
	OTA	4.0	0.20~0.85	Turkey	[31]
	AFB_1_	45.6	1.00~47.70	Italy	[29]
	AFB_1_ AFB_2_ AFG_1_ AFG_2_	40.0 30.0 40.0 20.0	5.30~10.15 1.46~3.47 1.90~3.31 0.81~0.90	Malaysia	[32]

AOH, Alternariol; TENT, Tentoxin.

**Table 3:** Contamination of mycotoxins in fruits, vegetables, and their products.

Product	Type of Mycotoxin	Detection Rate (%)	Contamination Value (μg/kg or μg/L)	Country	Reference
Apple	PAT	56.0	24.00~356.00	France	[36]
Tomato	TeA	100.0	11.00~4560.00	Italy	[37]
Green tea	AFB_2_ AFG_1_ ZEN ENB AOH TENT	2.0 2.0 35.0 2.0 40.0 1.0	3.66~7.40 1.35~1.60 14.32~45.80 0.10~0.30 1.70~5.90 0.31~4.60	Morocco	[38]
Sugarcane juice	AFs	22.2	0.50~6.50	India	[39]
Wine	OTA AOH PAT	47.0 52.0 32.0	0.57~2.28 0.61~26.86 15.35~88.24	Spain	[20]
Cava and cider	OTA PAT	26.0 40.0	0.77~2.44 14.73~41.93	Spain	[20]

ENB, Enniatin B; TeA, Tenu.

**Table 4:** Contamination of AFM_1_ in milk and products.

Product	Type of Mycotoxin	Detection Rate (%)	Contamination Value (μg/kg or μg/L)	Country	Reference
Liquid milk	AFM_1_	20.0	0.026~0.039	China	[43]
Raw milk	AFM_1_	58.8	0.01~0.44	Lebanon	[44]
	AFM_1_	6.0	0.008~0.15	Italy	[45]
Pasteurized milk	AFM_1_	91.0	5.30~85.20	China	[46]
	AFM_1_	40.0	0.00~1.21	India	[47]
Yoghurt	AFM_1_	59.0	10.00~66.70	China	[46]
	AFM_1_	64.3	0.015~0.545	Lebanon	[44]
	AFM_1_	30.0	0.00~0.30	India	[47]
UHT milk	AFM_1_	53.7	5.10~46.60	China	[46]
	AFM_1_	41.7	0.00~1.52	India	[47]
Milk	AFM_1_	67.0	0.001~23.10	Latin America	[48]

**Table 5:** Summary of recent studies on mycotoxins degradation in food with non-contact food processing technologies.

Target Mycotoxin	Treated Sample	Treatment Parameters	Degradation Effect	References
Gamma ray irradiation				
AFB_1_	Hazelnut	10 kGy 10 min	AFB_1_: 47.0%	[49]
AFM_1_	Milk	0.39 mGy per day	4 d: 51.5% 8 d: 99.0%	[50]
AFB_1_ OTA	Sorghum	10 kGy	AFB_1_: 59.0% OTA: 32.0%	[51]
AFB_1_	Peanut	5~9 kGy	20.0~43.0%	[52]
UV irradiation				
AFB_1_ OTA FB_2_	Wheat flour	30 W, 15 min	AFB_1_:100.0% OTA:100.0% FB_2_:100.0%	[53]
AFB_1_	Corn Peanut	10 d: 8.37 J·cm^−2^	Corn AFB_1_: 17.0~43.0% Peanut AFB_1_: 14.0~51.0%	[54]
AOH AME TeA	Tomato	2.5 J·cm^−2^	AOH: 44.5% AME: 37.1% TeA: 34.5%	[55]
AFB_1_ AFM_1_	Whole milk	0.836 J·cm^−2^ 0.857 J·cm^−2^	AFB_1_: 78.2% AFM_1_: 65.7%	[56]
AFB_1_	Peanut oil	3500 μW·cm^−2^ 120 s	AFB_1_: >95.0%	[57]
AFM_1_	Milk	UV-C_254 nm_, 5~20 min	20 min AFM_1_: 50.0%	[58]
PAT	Apple juice	45.06 J·cm^−2^	PAT: >98.0%	[59]
Electron beam irradiation				
OTA	Red pepper	30 kGy	OTA: 25.0%	[60]
AFs	Corn syrup	20 kGy	AFs decreases by 0.3 log (ng·g^−1^) on average	[61]
Microwave irradiation				
AFB_1_ OTA	Maize flour	2450 MHz 100% power	AFB_1_: 50.58% OTA: 46.97%	[62]
AFB_1_	Peanut	360 W, 6 min 480 W, 5 min 600 W, 3 min	AFB_1_: 59% AFB_1_: 67% AFB_1_: 62%	[52]
AFB_1_ AFB_2_ AFG_1_ AFG_2_ OTA	Pistachio	2450 MHz 100% power 10 min	AFB_1_: 34.6% AFB_2_: 23.3% AFG_1_: 29.3% AFG_2_: 36.6% OTA: 34.2%	[63]
Pulsed light				
DON	Barley	180 pulses in 60 s	DON: 69.1%	[64]
OTA	Grape juice	39 J·cm^−2^	OTA: 95.18%	[65]
PAT	Apple juice	24 J·cm^−2^	PAT: 74%	[66]
PAT	Apple juice	40.5 J·cm^−2^	PAT: >95.44%	[67]
AFB_1_ AFB_2_ AFG_1_ AFG_2_	Apple juice	40 flashes	AFB_1_: 71.96% AFB_2_: 73.32% AFG_1_: 54.04% AFG_2_: 69.58%	[68]
Pulsed electric field				
AFB_1_ AFB_2_ AFG_1_ AFG_2_	Sesame seed	17.28 J	AFB_1_: 86.9% AFB_2_: 98.7% AFG_1_: 94.7% AFG_2_: 92.7%	[69]
ENs BEA	Juices and Smoothies	30 kV, 3 kV·cm^−1^, 500 kJ·kg^−1^	43~70%	[70]
AFB_1_ AFB_2_ AFG_1_ AFG_2_	Grape juice	30 kV, 3 kV·cm^−1^, 500 kJ·kg^−1^	AFB_1_: 25% AFB_2_: 72% AFG_1_: 84% AFG_2_: 24%	[71]
AFB_1_ AFB_2_ AFG_1_ AFG_2_	Red pepper	17.28 J	AFB_1_: 97.75% AFB_2_:99.58% AFG_1_:99.88% AFG_2_:99.47%	[72]
DON T-2	Barley	Less intensive: 100 bipolar pulses, 10 Hz, 9 kV·cm^−1^ Intensive: 500 bipolar pulses, 10 Hz, 9 kV·cm^−1^	Less intensive: DON: 14% T-2: 18% Intensive: DON: 31% T-2: 24%	[73]
Plasma				
T-2 HT-2	Oat flour	Nitrogen: 100 Pa 30 min	T-2: 43.25% HT-2: 38.54%	[74]
DON	Wheat	100 V, 20 Hz, 15 min	DON: 61%	[75]
DON OTA	Rice grain	25 kV, 8 min	DON: 61.25% OTA: 55.64%	[76]
AFM_1_	Skim milk	200 W, 60 Hz, 20 min	AFM_1_: 78.9%	[77]
Ozone				
OTA	Sultanas	12.8 mg/L, 120/240 min	120 min:60.2% 240 min:82.5%	[78]
ZEN	Whole corn flour	51.5 mg/L 5, 60 min	5 min: 37.9% 60 min: 62.3%	[79]
AFB_1_ AFB_2_ AFG_1_ AFG_2_ DON OTA ZEN	Parboiled rice	5 L/min, 5 h	AFB_1_: 80.9% AFB_2_: 59.2% AFG_1_: 61.8% AFG_2_: 47.6% DON: 56.0% OTA: 87.9% ZEN: 75.9%	[80]
FB_1_ FB_2_	Corn kernel	13.5 mg·L^−1^, 1.0·L min^−1^, 24 h	FB_1_: 81.2% FB_2_: 86.2%	[81]

## Data Availability

No new data were created or analyzed in this study. Data sharing is not applicable to this article.
